# Peer-led problem-based learning in interprofessional education of health professions students

**DOI:** 10.3402/meo.v20.28851

**Published:** 2015-09-04

**Authors:** Michael D. Lehrer, Samuel Murray, Ruth Benzar, Ryan Stormont, Megan Lightfoot, Michael Hafertepe, Gabrielle Welch, Nicholas Peters, Anna Maio

**Affiliations:** 1 Office of Medical Education, Creighton University School of Medicine, Omaha, NE, USA; 2 Office of Medical Education, St Joseph's Hospital and Medical Center, Phoenix, AZ, USA

**Keywords:** interprofessional education, peer-teaching, problem-based learning, barriers, involvement, collaboration, undergraduate medical education, undergraduate pharmacy education, leadership

## Abstract

**Background:**

The role of peer teachers in interprofessional education has not been extensively studied. This study is designed to determine if peer-teacher-led problem-based seminars can influence medical and pharmacy students’ perceptions of interprofessional education.

**Methods:**

Undergraduate medical and pharmacy students participated in one-hour problem-based learning seminars held over the course of 16 weeks. A case–control study design was used to compare perceptions of interprofessional education between students who participated in seminars and students who did not participate in seminars. The validated Interdisciplinary Education Perception Scale (IEPS) was used to assess perceptions of interprofessional education and was distributed to medical and pharmacy students at the conclusion of 16 weeks of seminars. A two-tailed *t*-test was used to determine significance between groups. A survey was also distributed to all students regarding perceived barriers to involvement in interprofessional education training.

**Results:**

In total, 97 students responded to IEPS (62 medical, 35 pharmacy). Data showed significantly higher perception of professional cooperation among medical students (*p=*0.006) and pharmacy students (*p=*0.02) who attended interprofessional seminars compared to those who did not attend. One hundred and nine students responded to the survey regarding perceived barriers to interprofessional education, with the two most common barriers being: ‘I am not aware of interprofessional education opportunities’ (61.5%) and ‘I do not have time to participate’ (52.3%).

**Conclusion:**

Based on this data we believe peer-teacher-led problem-based interprofessional seminars can be used to increase medical and pharmacy students’ perceived need for professional cooperation. Currently, major barriers to interprofessional education involvement are awareness and time commitment. Undergraduate health professions education can incorporate student-led seminars to improve interprofessional education.

Optimal patient care relies on a cohesive team of health care professionals who work synergistically, focusing on individual strengths to ensure the highest quality of patient care (1, 2). It has been shown that interprofessional collaboration enhances patient safety, decreases medical errors, and improves satisfaction among health professionals (2, 3). Skills necessary to function as part of an interprofessional team require cultivation and development during health professions training (4). This has made training in interprofessional collaboration an increasingly appreciated component of medical education.

Interprofessional education (IPE) occurs when two or more professions learn with, from, and about each other to improve collaboration and the quality of care (5). The development and implementation of interprofessional education curriculum, however, can be a difficult task, requiring significant faculty involvement and curriculum development (6).

Student-led peer teaching conveys a number of benefits to the peer learner, the peer teacher, and the institution as a whole (7, 8). Peer teaching occurs when instructors and learners are at a similar stage in their education (9). Students engaged in peer learning have been shown to be highly receptive to instruction from peer teachers due to the decreased power differential existing between peer teacher and peer learners (10). In addition, student-led initiatives are cost-effective and can promote collegiality and socialization, leading to long-term sustainability of student-led programs (8).

Small-group problem-based learning (PBL) is a highly effective means of education (11). During PBL sessions students are presented with a problem or clinical scenario and are then required to work together to come up with a solution with minimal input from the facilitator (12). The problem-based model is designed around individual inquiry and is very effective in developing problem solving skills, independent learning, and teamwork (13). This educational method presents a unique way of delivering IPE. We believe student-led small-group PBL seminars can be used to effectively deliver IPE and improve students’ perceived need for interprofessional education.

To measure the efficacy of IPE sessions, the Interprofessional Education Perception Scale (IEPS) tool was used (14). The IEPS tool, originally developed by Leucht et al., is an 18-item questionnaire using a six-point Likert scale, designed to assess perceptions of interprofessional education. The questionnaire is then subdivided into four subscales of IPE: competence and autonomy (Subscale 1), perceived need for cooperation (Subscale 2), perception of actual cooperation (Subscale 3), and understanding of others values (Subscale 4). The IEPS was used to evaluate participants’ opinions regarding IPE over 16 weeks (Table 1).

**Table 1:** IEPS subscale classifications

Subscale 1	Professional competence and autonomy
Subscale 2	Perceived need for professional cooperation
Subscale 3	Perception of interdependence
Subscale 4	Willingness to share ideas

## Methods

First- and second-year medical and pharmacy students at Creighton University were invited to participate in small-group PBL interprofessional seminars led by fellow second-year medical students who served as peer-teachers. The study was approved by the Institutional Review Board at Creighton University.

Seminars were held weekly during the lunch hour for 16 weeks. Each seminar was led by one to two second-year medical students, serving as peer-teachers and consisted of 10–14 student learners, split evenly between medical and pharmacy students. The cases were adaptations of cases seen at the Creighton University Medical Center or cases published in peer-reviewed journals. At the beginning of each seminar the group was given a basic patient presentation. Student learners were then required to ask appropriate questions and find appropriate answers to create a differential diagnosis. Student learners were next asked to identify appropriate diagnostic testing to order. The designated test results and questions regarding history and physical were provided by the peer-teacher when appropriate. Through the course of the seminar the peer-teacher provided little to no guidance in keeping with the PBL design. During each seminar students were allowed to use any reference material they deemed necessary.

## Evaluation

A case–control study design was used to evaluate the efficacy of IPE seminars. Following 16 weeks of IPE seminars, student's attitudes toward IPE were assessed using the IEPS. Questionnaires were distributed to the entire first- and second-year medical and pharmacy student classes, including those who did not attend. Those students not attending the IPE seminars served as the control group for our study. All first- and second-year medical and pharmacy students were also given a questionnaire regarding barriers to IPE; this survey was designed by the authors of this study (Table 2).

**Table 2:** IEPS scaled results

	*n*	Subscale 2 mean±SD	Subscale 3 mean±SD
All medical students	62	94.76^a^±6.44	75.91^b^±10.32
All pharmacy students	35	90.24^a^±10.00	84.29^b^±10.05
Medical student participants	19	97.81^c^±4.68	76.67±11.65
Medical student non-participants	43	93.45^c^±7.02	74.76±10.04
Pharmacy student participants	10	95.37^d^±5.71	86.33±10.82
Pharmacy student non-participants	25	88.33^d^±10.76	82.67±10.93

a All medical students compared to all pharmacy students ‘need for professional cooperation’ *p*=0.02

b all medical students compared to all pharmacy students ‘perception of interdependence’ *p*=0.002

c medical student participants compared to medical student non-participants ‘need for professional cooperation’ *p*=0.006

d pharmacy student participants compared to pharmacy student non-participants ‘need for professional cooperation’ *p*=0.02.

Data from the IEPS were analyzed along four subscales as described previously (14). The primary outcome was the difference in responses to the IEPS of those students who attended seminars versus those students who did not attend. The data were scaled to a score of 100 for easier interpretation. It is important to note that data can only be compared within each subscale and not between subscales. Statistical significance was determined using two-tailed *t-*test and a *p* value of 0.05. Student responses with regard to perceived barriers to interprofessional education were also recorded and served as secondary outcome data.

## Results

In total, 43 students (24 medical and 19 pharmacy) attended IPE seminars, of those 29 completed the IEPS (67.4% response rates). Sixty eight (43 medical and 29 pharmacy) out of 313 students who did not participate in IPE seminars completed the IEPS survey (21.7% response rate) (Table 2). IEPS responses show that medical and pharmacy students who participated in seminars perceived a significantly higher need for cooperation when compared to those who did not participate (*p=*0.006 and *p=*0.02, respectively). Significance was not observed within subscale 1 or subscale 4 (data not shown). In addition, combined responses from participants and non-participants showed that pharmacy students perceived a significantly higher need for professional cooperation (*p=*0.02) and interdependence (*p=*0.002) when compared to medical students.

In total, 109 (30.62%) out of 356 eligible students responded to the survey regarding current barriers to interprofessional education (Table 3). The most common barrier to participation was ‘I am not aware of interprofessional education programs’ (61.5% of respondents). The second most common response was ‘I do not have time’ (52.3% of respondents). Zero respondents indicated, ‘Interprofessional education is not an important part of my career’.

**Table 3:** Student perceived barriers to IPE

Student response	%	*n*
I am interested in IPE but I do not have enough time to participate	52.3	57
I am not aware of currently available IPE activities	61.5	67
I have tried and all IPE sessions are full	5.5	6
I do not think IPE is important in my future	0.0	0

## Discussion

The role that student leaders play in curricular development, especially pertaining to interprofessional education, can be of great value to educational institution as a whole. Current student-led initiatives in IPE have used designs such as social gatherings, conferences, lectures, and formal communications between health professions students (8). Our data demonstrate that a student-led small-group PBL seminar can be used to effectively address certain IPE goals. In particular, students in our study who participated in IPE seminars demonstrated significantly greater perceived need for interprofessional cooperation when compared to those who did not attend.

In addition, the most commonly cited barrier to involvement in IPE in our study was lack of awareness of IPE programs. In our case, the lack of awareness of IPE programs is due to the absence of IPE in the standard curriculum. IPE programs can be resource and time intensive because of the significant amount of coordination required to educate students in different health professions (15). Peer-teachers are a significantly underutilized resource available to educators, and can be used to effectively achieve educational outcomes that may otherwise be difficult based on funds or faculty availability (16, 17). Seminars in our study were designed and led exclusively by medical students and were minimally resource intensive on faculty and school funding.

Students also state that time is a major barrier to participation in interprofessional education. Minimal preseminar preparation and the relaxed nature of peer-led seminars significantly reduce the time constraint that students feel in more formal educational environments. These seminars fit nicely into a lunch hour, similar to noon-conference seminars used in many graduate medical education training programs.

The use of small-group PBL sessions has been shown to be highly effective in developing problem solving skills and teamwork (13). During our study it was evident that students frequently encountered problems they could not solve on their own and required the help of students from other health professions to solve such problems. This interprofessional reliance began to shape the attitudes and behaviors of students in our study and resulted in appreciation of the interprofessional collaboration required to optimally approach a patient or clinical problem. Other studies have observed similar findings using a broad range of IPE models (17).

There are certain limitations to this study; in particular our study was limited to pharmacy and medical students based on logistical constraints. It is yet to be proven whether these results are applicable to other health professions. Also, the voluntary nature of participation may select for those students who inherently desire growth in interprofessional collaboration, thus further studies are required to control for this variable and determine the generalizability to an entire student population.

One of the most encouraging observations of this study was the vigor with which new students approached leadership roles within IPE seminars. Because of the progressive nature of medical education it is crucial that new leaders emerge who can effectively continue programs and improve upon previous iterations (8). The minimal faculty involvement and complete student autonomy over IPE seminars seemed to inspire peer-learners to become peer-teachers.

## Conclusion

In conclusion, peer-teacher-led problem-based interprofessional seminars are effective in improving the perceptions of interprofessional collaboration among first- and second-year medical and pharmacy students. The peer-teaching model may be an effective adjunct to traditional curriculum in addressing IPE by overcoming major barriers to student involvement.
